# Complete genome of marine flavobacterium *Dokdonia sp*. strain Dokd-P16 isolated from the low-iron waters of the subarctic Northeast Pacific Ocean

**DOI:** 10.1128/mra.00866-25

**Published:** 2026-04-20

**Authors:** Anwar Alqattan, Curtis A. Suttle, Maria T. Maldonado

**Affiliations:** 1 Department of Earth, Ocean and Atmospheric Sciences, The University of British Columbia 8166, Vancouver, British Columbia, Canada; 2 Department of Microbiology and Immunology, University of British Columbia 8166, Vancouver, British Columbia, Canada; 3 Department of Botany, University of British Columbia 8166, Vancouver, British Columbia, Canada; 4 Institute for the Oceans and Fisheries, University of British Columbia 8166, Vancouver, British Columbia, Canada; California State University San Marcos, San Marcos, California, USA

**Keywords:** heterotrophic bacterium, *Dokdonia*, iron limitation, flavobacterium, proteorhodopsin

## Abstract

The genome of *Dokdonia* sp. strain Dokd-P16, isolated from iron-limited waters of the subarctic Northeast Pacific Ocean, shows evidence of adaptation to iron/nutrient-limiting conditions. These include coding sequences for two putative rhodopsins and for proteins involved in iron acquisition and homeostasis, gliding motility and hydrolysis of complex carbohydrates.

## ANNOUNCEMENT

Most members of phylum Bacteriodota carry putative genes for gliding motility and hydrolytic enzymes to degrade algal polymers under nutrient-limited conditions (1–3). Trace metals, like iron, influence their genomic traits that rely on iron-containing proteins for biochemical processes (4).

The proteorhodopsin-containing gram-negative, marine heterotrophic flavobacterium *Dokdonia* sp. strain Dokd-P16 was isolated from the iron-limited surface layers at Station P16 (49°17.00 and 134°40.00, Cruise, 22 May–9 June 2012) in the Northeast (NE) Pacific Ocean, which is a part of the high-nutrient, low-chlorophyll region, well-established as iron-limited sites through enrichment bioassays, dissolved iron measurements, and regional mesoscale iron-enrichment experiments (5–9).

Samples were serially diluted up to 10⁻^5^ and plated on marine agar containing 0.05 g L⁻¹ of a 1:1 mix of bactopeptone and casein hydrolysate (6, 7), prepared in 0.22 μm filtered seawater collected from Station P16. Colonies were subsequently purified by quadrant streaking onto CMP agar plates and incubation at 19 ± 1°C for 24–48 h (6, 7). Pure cultures were inoculated to CMP liquid medium and incubated at 19 ± 3°C for 24 h in light, without shaking. Genomic DNA was extracted using phenol–chloroform method with minor modifications (10). Cells were treated with lysozyme (5 mg/mL, 37°C, 30 min), followed by 1% SDS and proteinase K (200 µg/mL, 55°C, 1 h) and RNase A (100 µg/mL, 37°C, 2 h). DNA was purified using phenol:chloroform:isoamyl alcohol (25:24:1) with chloroform re-extraction and precipitated with 0.5 volume 7.5 M ammonium acetate and two volumes isopropanol (−20°C, 1 h).

**Fig 1:**
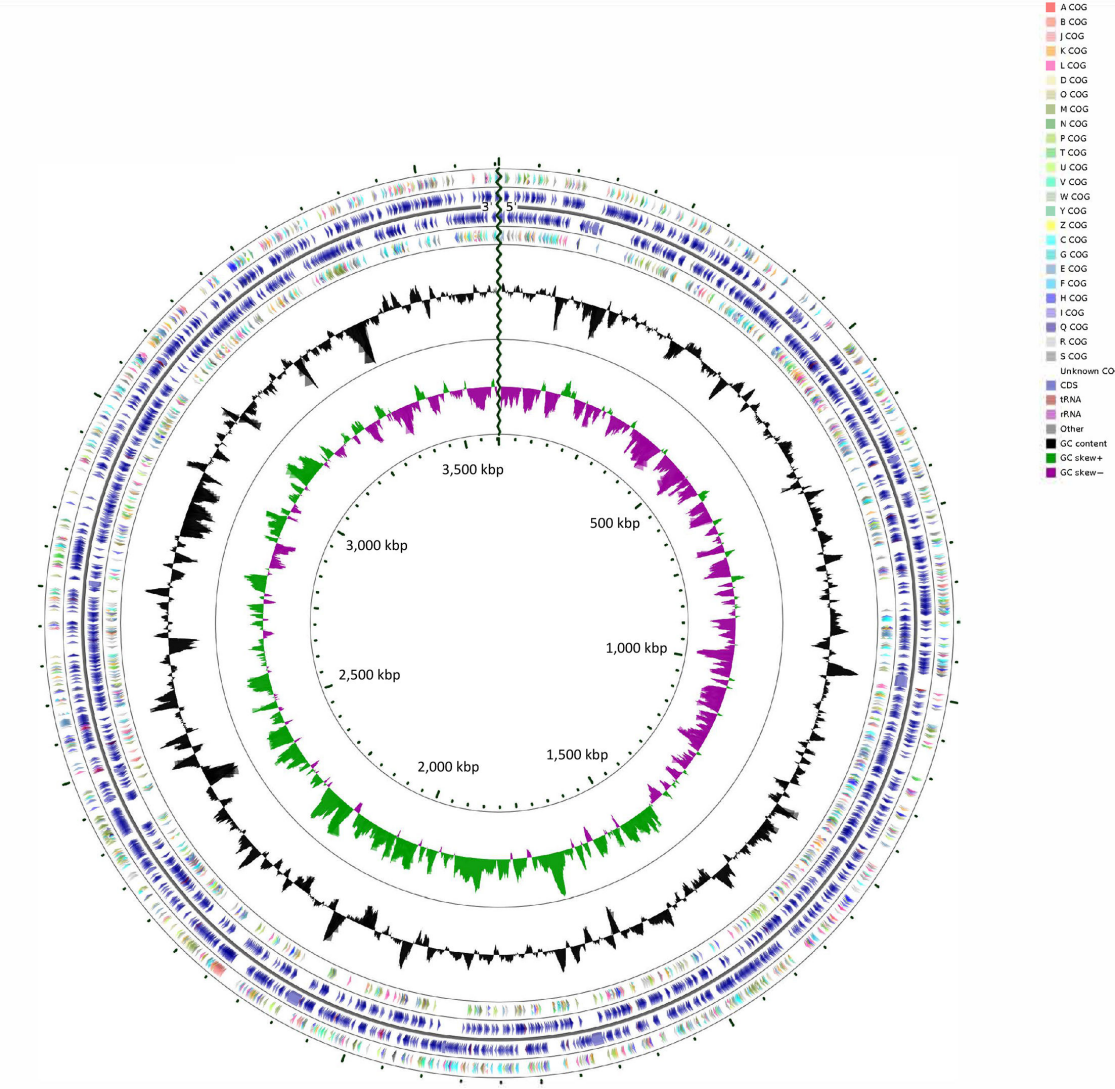
Circular genomic map of *Dokdonia* sp. strain Dokd-P16 generated using the CGview server. The outermost to innermost rings of the map represent the following: (i) Clusters of Orthologus Groups (COG) functional categories for forward strand coding sequences; (ii) forward strand sequence representing the locations of identified genes; (iii) reverse strand sequence representing locations of identified genes; (iv) COG functional categories for reverse strand coding sequences; (v) the black ring shows GC content; and (vi) GC skew, with the green and purple bands representing positive and negative values, respectively.

Ten micrograms of genomic DNA was sequenced using Pacific Biosciences RSII sequencer (University of Delaware Sequencing and Genotype Center, USA). Samples were prepared using the SMRT bell Template Prep Kit 1.0 (PacBio), and size selection was performed using BluePippin (Sage Science, USA) with a 10 kb cutoff. PacBio generated a total of 96,700 reads, and raw reads were processed using the SMRT Analysis pipeline, to remove adapter sequences and filter subreads, resulting in 40,583 reads with an N50 read length of 15,235 bp. The genome was assembled using PacBio’s HGAP (ver3) (10) with no detectable plasmids. Additionally, *de novo* assembly using Canu (v2.1.1) identified overlapping contig ends, and the chromosome was circularized using Circlator v1.4.1 (11, 12). The Dokd-P16 consists of a circular double-stranded genome of 3,623,447 bp, with an average Guanine-Cytosine (GC) content of 37.16% (Fig. 1). CheckM (Ver 1.1.3) analysis showed a completeness of 99.24% with zero contamination (13) (Table 1).

**TABLE 1:** General features of *Dokdonia sp.* strain Dokd-P16 genome in comparison to other *Dokdonia* species

Parameters	*Dokdonia* sp. Dokd-P16	*Dokdonia* sp. 4H-3-7-5	*Dokdonia* sp. PRO95	*Dokdonia donghaensis* MED134	*Dokdonia donghaensis* DSW-1	*Dokdonia pacifica* DSM 25597	*Dokdonia sinensis* SH27	*Dokdonia* sp. Hel_I_63
Size (bp)	3,623,447	3,389,993	3,305,093	3,301,953	3,219,590	5,520,745	3,651,050	3,472,909
% G + C	37.16%	37.29%	37.38%	38.16%	38.07%	34.10%	39.53%	37.26%
Predicted genes	3,202	3,048	3,038	2,999	2,923	4,968	3,325	3,130
Predicted no. of Coding DNA sequence (CDS)	3,146	2,995	2,978	2,944	2,865	4,884	3,274	3,038
RNA genes	56	53	60	55	58	84	46	92
Ribosomal RNA	9	9	2	9	5	6	3	17
Transfer RNA	43	42	36	46	40	68	39	49
Other RNA	3	2	22	NA	13	10	4	26
Genes coding signal peptides	425	348	396	391	406	702	501	374
GenBank accession number	CP029151	CP002528	CM001837	CP009301	CP015125	FZNY00000000	REFV00000000	VISD00000000
Completeness value	99.24	99.62	98.86	99.62	99.62	99.62	99.62	99.62
Isolation	Surface seawater (25 m depth, Station P-16, NE Pacific Ocean)	Subseafloor sediments (31.4 m depth, Suruga Bay, Japan)	Surface seawater (Hogland island, North Sea, Germany)	Surface seawater (0.5 m depth, Northwest Mediterranean Sea)	Seawater (in between the two islands of Dokdo, East Sea)	Surface seawater (South Pacific Gyre)	Seawater (Xiaoshi Island, China)	(Hogland island, North Sea, Germany)
References	(5)	(14)	(15)	(16)	(17)	(18)	(19)	–^*a*^

^*a*^ –, not applicable.

The genome was annotated using National Center for Biotechnology Information (NCBI) Prokaryotic Genome Annotation Pipeline (v.4.5) (20), identifying 3,202 open reading frames and 3,119 protein coding genes (Table 1). Coding genes and metabolic pathways were annotated using Kyoto Encyclopedia of Genes and Genomes (21) and RAST Annotation Server and Pathway Tools software (Ver 2) (22–24). Pfam (Ver 32.0) (25) analysis assigned functional annotations to 75.8% of the protein-coding genes, with 6.2% being involved in coenzyme transport and metabolism, and 5.8% in inorganic ion transport and metabolism. Furthermore, 17 regulatory and miscellaneous elements were assigned, and 927 hypothetical genes were identified without functional predictions using PHASTER (26). All analyses were performed using default parameters.

The Dokd-P16 genome encodes a full set of genes for gliding motility, ABC/TBDT receptors for efficient iron and DOM uptake, and *de novo* vitamin B1 synthesis. In contrast, it lacks genes for iron-dependent enzymes such as Fe-SOD and FumA, suggesting adaptation to nutrient/iron-limited conditions.

## Data Availability

This genome project is registered in GenBank under BioProject; PRJNA450898, the complete genome and 16S ribosomal RNA sequences are available in GenBank (NZ_CP029151.1 and PQ870337, respectively). All raw sequencing files are deposited in NCBI Sequence Read Archive database under SRR33419195. Genome annotation details are available on Figshare (https://doi.org/10.6084/m9.figshare.29816336 and https://doi.org/10.6084/m9.figshare.29850293).
